# Managing Ocular Surface Disease in Glaucoma Treatment: A Systematic Review

**DOI:** 10.3390/bioengineering11101010

**Published:** 2024-10-11

**Authors:** Özlem Evren Kemer, Priya Mekala, Bhoomi Dave, Karanjit Singh Kooner

**Affiliations:** 1 Department of Ophthalmology, University of Health Sciences, Ankara Bilkent City Hospital, Ankara 06800, Turkey; ozlemvidya@gmail.com; 2 Department of Ophthalmology, University of Texas Southwestern Medical Center, Dallas, TX 75390, USA; priya.mekala@utsouthwestern.edu (P.M.); bhd27@drexel.edu (B.D.); 3 Drexel University College of Medicine, Philadelphia, PA 19129, USA; 4 Department of Ophthalmology, Veteran Affairs North Texas Health Care System Medical Center, Dallas, TX 75216, USA

**Keywords:** ocular surface disease, glaucoma, topical medications, preservatives, benzalkonium chloride

## Abstract

Ocular surface disease (OSD) is a frequent disabling challenge among patients with glaucoma who use benzalkonium chloride (BAK)-containing topical glaucoma medications for prolonged periods. In this comprehensive review, we evaluated the prevalence of OSD and its management, focusing on both current and future alternatives. Preferred Reporting Items for Systematic Reviews and Meta-Analyses (PRISMA) criteria were used to assess a) the impact of active ingredients and preservatives on the ocular surface and b) the efficacy of preservative-free (PF) alternatives and adjunctive therapies. BAK-containing glaucoma medications were found to significantly contribute to OSD by increasing corneal staining, reducing tear film stability, and elevating ocular surface disease index (OSDI) scores. Transitioning to PF formulations or those with less cytotoxic preservatives, such as Polyquad^®^ and SofZia^®^, demonstrated a marked improvement in OSD symptoms. In particular, the use of adjunct cyclosporine A, through its anti-inflammatory and enhanced tear film stability actions, was shown to be very beneficial to the ocular surface. Therefore, the most effective management of OSD is multi-factorial, consisting of switching to PF or less cytotoxic medications, adjunct use of cyclosporine A, and early incorporation of glaucoma surgical treatments such as laser trabeculoplasty, trabeculectomy, glaucoma drainage devices, or minimally invasive glaucoma surgery (MIGS).

## 1. Introduction

Glaucoma, a global multi-factorial disease, is characterized by progressive degeneration of the optic nerve with or without elevated intraocular pressure (IOP). It is the most common cause of irreversible blindness, and its global prevalence is estimated to be around 4% in patients between the ages of 40 and 80 years [1]. Topical medical therapy has been most commonly used for many years. Research indicates that the average number of medications prescribed is 3.09 and eye drops form the bulk of therapy [2]. This chronic use of multiple topical drugs, combined with other factors such as age and systemic comorbidities and their treatments, profoundly contributes to ocular surface disease (OSD).

**Figure 1:**
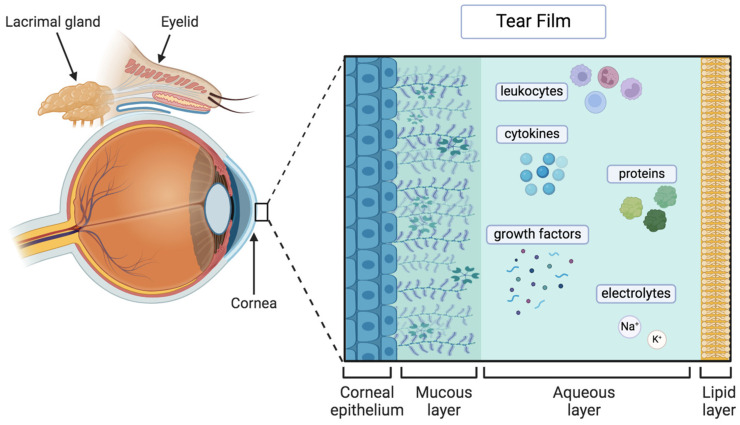
Representation of the ocular surface and tear film composition (corneal epithelium, mucous layer, aqueous layer, and lipid layer). (Figure made using BioRender^®^ software, version 201 and adapted from [9]).

**Figure 2:**
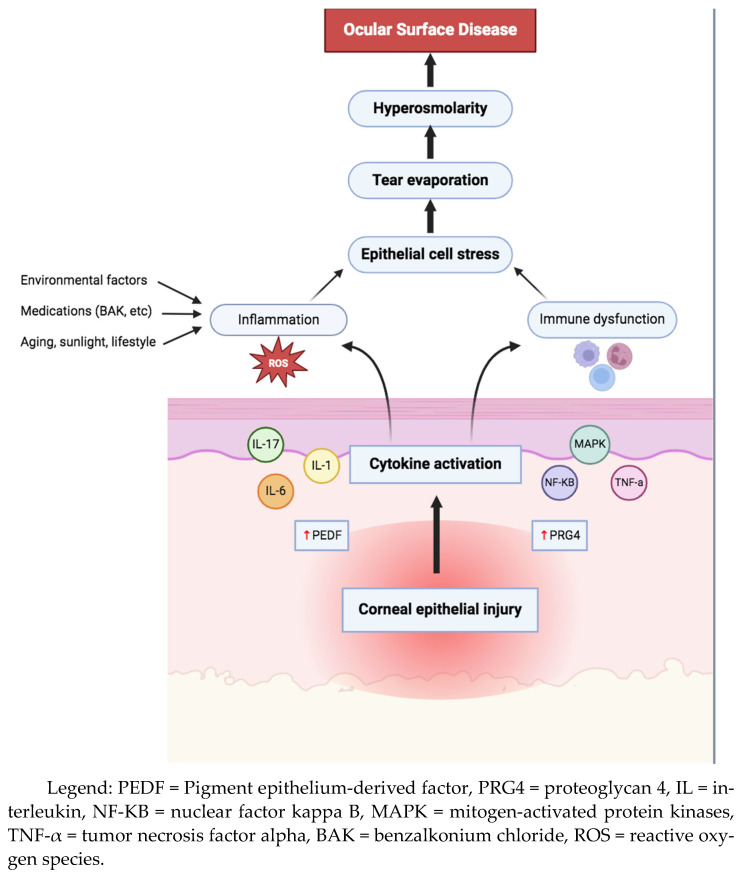
A brief overview of the immune-inflammatory mechanisms in the pathogenesis of ocular surface disease (Figure made using BioRender^®^ software, version 201).

**Figure 3:**
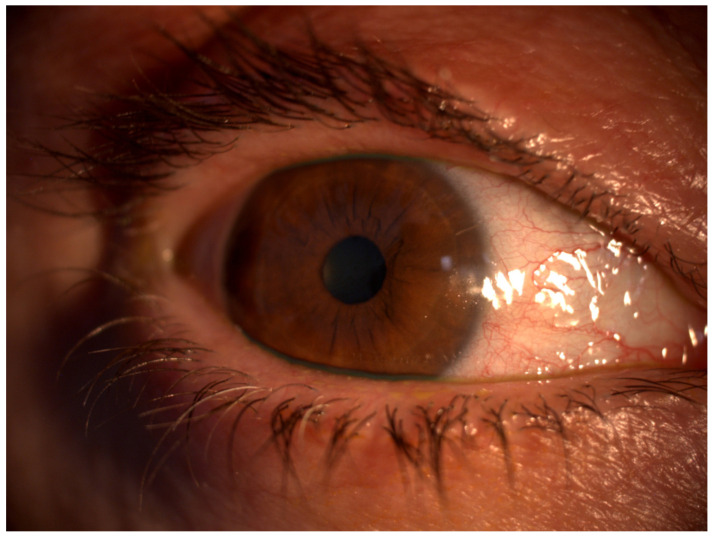
External photograph of an eye with OSD showing MGD, blepharitis, and conjunctival hyperemia. Image courtesy of Karanjit S. Kooner, MD, PhD (University of Texas Southwestern Medical Center, Dallas, TX, USA).

**Figure 4:**
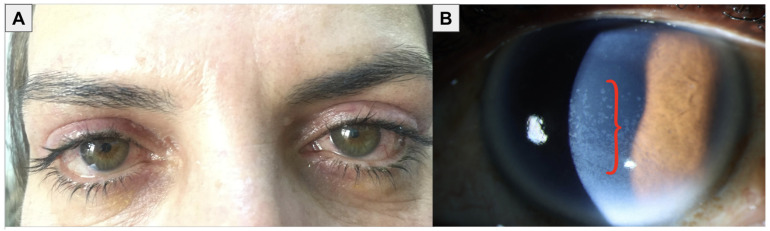
Clinical photographs of ocular surface disease. (**A**) External photograph of a patient with chronic hyperemia and MGD. (**B**) Slit lamp photograph of an eye with superficial punctate keratitis (red curly bracket). Images courtesy of Özlem Evren Kemer, MD (Ankara Bilkent City Hospital, Ankara, Turkey) and Margaret Wang French, MD (University of Texas Southwestern Medical Center, Dallas, TX, USA).

OSD is a complex condition that impacts both the tears and the ocular surface (Figure 1), leading to various symptoms such as discomfort, visual disturbances, and tear film instability [3]. It is characterized by increased tear film osmolarity and inflammation, which can manifest clinically as superficial punctate keratitis (SPK), conjunctival hyperemia, and papillary conjunctivitis (Figure 2). The etiology of OSD is diverse and includes environmental and genetic factors, aging, dry eye syndrome, blepharitis, meibomian gland dysfunction (MGD), and the chronic use of eye drops with preservatives [4] (Figure 3 and Figure 4). Thus, OSD has broader implications than dry eye disease (DED) alone [5,6].

Ocular surface inflammation is thought to play a key role in the pathogenesis of OSD [3]. A 2020 meta-analysis by Roda et al. analyzed 13 articles involving 342 patients with DED and 205 healthy controls. Their systematic review revealed that DED patients had higher levels of tear interleukin (IL)-1β, IL-6, IL-8, IL-10, interferon-γ (IFN-γ), and tumor necrosis factor-α (TNF-α) compared to controls [7]. However, the Dry Eye Assessment and Management (DREAM) study, which analyzed 131 patient tear samples for various tear cytokines levels, including IL-1β, IL-6, IL-8, IL-10, IL-17A, IFNγ, and TNFα, found that only cytokines IL-10, IL-17A, and IFNγ were highly correlated with each other but weakly correlated with some DED signs [8].

Research indicates that 48–59% of patients with glaucoma experience symptoms of OSD, while 22–78% may exhibit clear clinical signs [10,11]. The long-term use of glaucoma medications, especially those containing benzalkonium chloride (BAK), often exacerbates OSD, leading to decreased quality of life, reduced adherence to treatment, and diminished therapeutic efficacy [12]. Therefore, prompt and effective management of OSD is paramount to maintaining treatment effectiveness, considering the higher prevalence of pre-existing dry eyes in this age group [12].

### 1.1. Diagnosis of OSD

**Figure 5:**
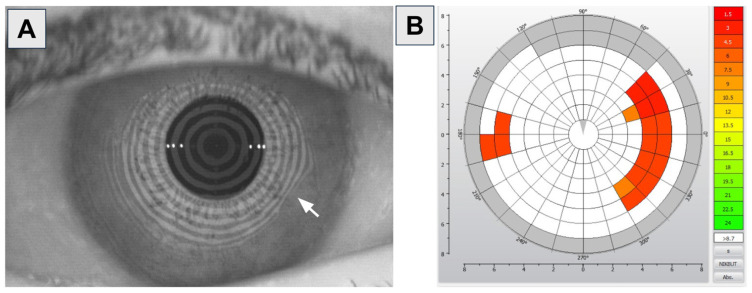
Keratograph of an eye with OSD. (**A**) Keratograph of an eye with areas of dryness (arrow) disrupting the placido disk reflections on the cornea. (**B**) Red-orange areas correspond to faster NITBUT. (OCULUS Keratograph^®^, OCULUS, Wetzlar, Germany). Images courtesy of Karanjit S. Kooner, MD, PhD (University of Texas Southwestern Medical Center, Dallas, TX, USA).

**Figure 6:**
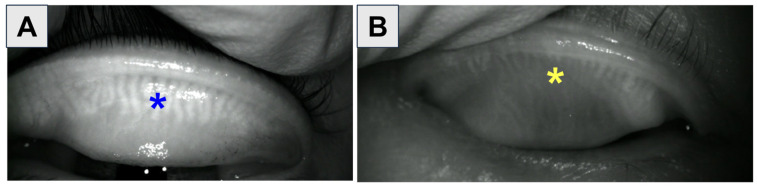
Clinical examples of meibography. (**A**) Meibography in a patient with healthy meibomian glands (asterisk). (**B**) MGD with significant atrophy of meibomian glands with ghosting (pale glands with abnormal meibomian gland architecture, asterisk). Images courtesy of Karanjit S. Kooner, MD, PhD (University of Texas Southwestern Medical Center, Dallas, TX, USA).

Various clinical tests, symptom questionnaires (Ocular Surface Disease Index [OSDI]), and imaging modalities are utilized in the diagnosis of OSD. Common clinical tests include Schirmer’s test, invasive tear break-up time (TBUT), fluorescein staining, and lissamine green staining [13]. The OSDI questionnaire consists of 12 questions (three for ocular symptoms, six for vision-related functions, and three for environmental triggers). The scores range from 0 to 100, with higher values corresponding to a greater impact on a patient’s daily life: 0–12, normal; 13–22, mild; 23–32, moderate; and 33–100, severe. Various corneal imaging devices can provide information regarding the tear meniscus height (TMH), non-invasive tear break-up time (NITBUT), and meibography [14]. Unlike invasive TBUT, NITBUT measurements are performed without fluorescein dye, utilizing videokeratoscopy to detect variations in the placido disks that are reflected on the cornea (Figure 5) [15]. Meibography evaluates the meibomian glands in vivo and a meiboscore can be calculated to quantify loss of meibomian glands (Figure 6).

### 1.2. Previous Research

Previous literature reviews describe adverse effects of anti-glaucoma medications on various ocular and periocular structures, mention the effects of some active ingredients and preservatives on the ocular surface, and outline some emerging medication delivery systems [16,17]. However, few studies exist that clearly and thoroughly describe the topical complications of each active ingredient and preservative present in anti-glaucoma treatments and provide a broad overview of the major innovations and future directions.

The purpose of this systematic review is to assess a) the impact of active ingredients and preservatives of anti-glaucoma treatments on the ocular surface and b) the efficacy of preservative-free (PF) alternatives and adjunctive therapies. The article also includes an overview of the future directions and novel therapies in the management of OSD in patients using topical glaucoma medications.

## 2. Materials and Methods

### 2.1. Initial Search

Our study was approved by the institutional review board of Ankara Bilkent City Hospital and exempted from full review as no patient information was used. We followed the Preferred Reporting Items for Systematic Reviews and Meta-Analyses (PRISMA) guidelines during data collection and the PICOS (Population, Intervention, Comparison, Outcomes, and Study) framework to create eligibility criteria, Table 1, [18]. The following keywords and MeSH terms were used: “glaucoma” (or “glaucoma, angle-closure”, “glaucoma, open-angle”), “dry eye syndromes”, “ocular surface disease”, “antiglaucoma agents” (or “ophthalmic solutions”), and “preservatives, pharmaceutical” (or “benzalkonium compounds”).

**Figure 7:**
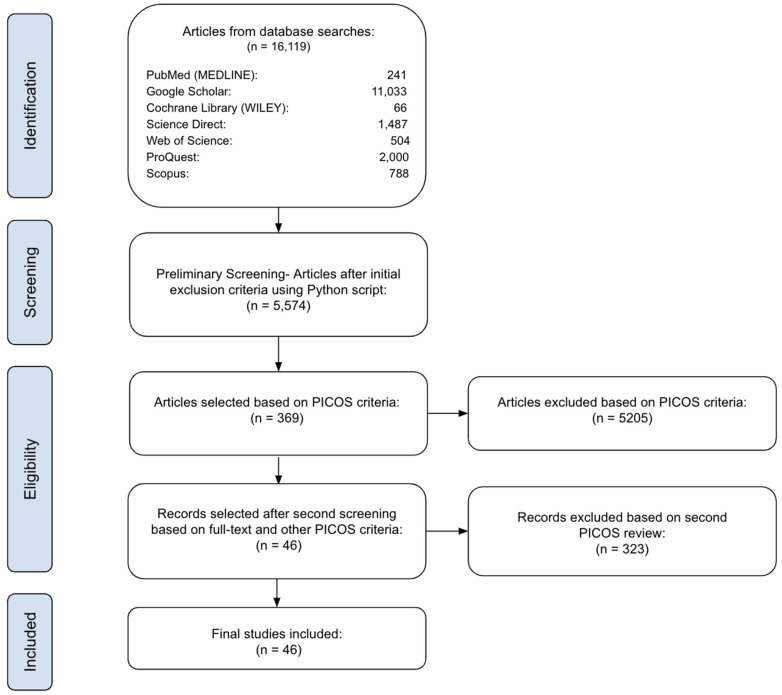
PRISMA flow chart.

Utilizing these keywords and MeSH terms, we systematically searched the online databases of PubMed (MEDLINE), Cochrane Library (Wiley), ScienceDirect, Scopus, Google Scholar, ProQuest, and Web of Science up to 20 July 2024. Comma-separated values (CSV) or Microsoft Excel files (Microsoft^®^ Excel, Redmon, WA, USA, version 16.87) were downloaded directly from each database. Considering Google Scholar search results, they were downloaded in CSV format utilizing the Publish or Perish software program (Anne-Wil Harzing, London, England, version 8.12.4612) [19]. All citations were then compiled in a single CSV file. There was a total of 16,119 articles obtained through this preliminary search (Figure 7).

### 2.2. Preliminary Screening

We excluded duplicates, non-English language articles, conference abstracts, and commentaries using a Python script (Python Software Foundation, Wilmington, DE, USA, version 3.12.2). The remaining articles were stored in a single CSV and contained author names, title, date of publication, journal name, and digital object identifier (DOI). A total of 5574 articles remained after preliminary screening.

### 2.3. Eligibility Assessment

Each article in the CSV was screened utilizing the PICOS criteria mentioned in Table 1, focusing on full-text English articles and studies involving animal or human subjects. After careful screening, an initial 369 articles was finally reduced to 46.

## 3. Results

Out of 16,119 articles initially identified, only 46 qualified for our final review based on our strict criteria.

### 3.1. Active Ingredients

There are multiple anti-glaucoma medications available, and they act via different pathways (Table 2). The active ingredients in them may directly irritate and disrupt the ocular surface via several mechanisms, such as toxicity to corneal epithelium leading to cytokine activation, inflammation, immune system dysfunction, epithelial cell stress, tear evaporation, and hyperosmolarity, contributing to the symptoms of OSD (Figure 2). The main clinical studies examining the side effects of glaucoma medications in particular OSD are shown in Table 3.

#### 3.1.1. Beta-Adrenergic Blockers

**Figure 8:**
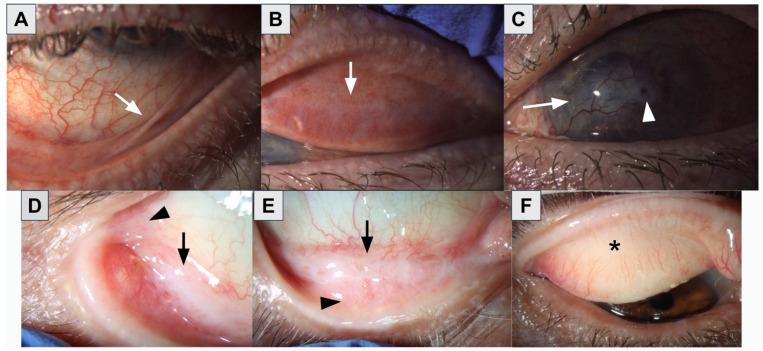
Two patients with ocular cicatricial pemphigoid ((**A**–**C**) patient 1) and ((**D**–**F**) patient 2). (**A**) symblepharon (arrow); (**B**) supratarsal conjunctival scarring (arrow); (**C**) corneal scarring, neovascularization (arrow), and healed descemetocele (arrowhead); (**D**) symblepharon (arrow) and subconjunctival fibrosis (arrowhead); (**E**) symblepharon (arrow), subepithelial fibrosis (arrowhead), inferior forniceal shortening; (**F**) meibomian gland dropout with subepithelial fibrosis (asterisk). Images courtesy of Karanjit S. Kooner, MD, PhD (University of Texas Southwestern Medical Center, Dallas, TX, USA) and Özlem Evren Kemer, MD (Ankara Bilkent City Hospital, Ankara, Turkey).

Topical beta-adrenergic blockers reduce aqueous humor (AH) and tear production by blocking beta receptors both on the ciliary epithelium and the main and accessory lacrimal glands [50]. In addition, their sympathomimetic activity may interfere with the epithelial cell viability/homeostasis. Thus, they have several side effects, such as a decrease in tear volume, MGD, conjunctival goblet cell loss, pseudo-pemphigoid cicatrizing conjunctivitis (Figure 8), and nasolacrimal duct obstruction [5,20].

Kuppens et al. reported that the TBUT decreased significantly in patients using both preserved (P) and timolol-PF in comparison to the control group. Thus, timolol-PF and P timolol formulations may both alter the tear film [21]. Other studies have shown that topical beta-blockers may also interfere with the corneal epithelium by inhibiting the sympathetic activity of limbal stem cells, resulting in SPK [22,23]. Laser scanning confocal microscopy and impression cytology have both revealed that beta blockers are toxic to the limbal stem cell microenvironment, thereby delaying corneal epithelial regeneration [23].

In 2003, a Japanese study involving 110 patients with glaucoma (35–88 years with mean age 69.7 ± 10.8) found that SPK was observed in 29.0% of cases [24]. Timolol users had a significantly higher occurrence of SPK (46.2%) compared to those using carteolol (4.2%). Interestingly, the prevalence of SPK was higher in patients using more than two anti-glaucoma eye drops (35.9%) compared to those using no eye drops (19.7%) or only one eye drop (30.9%). Notably, PF timolol still caused tear instability, suggesting that the active ingredient may damage the ocular surface [25]. A cross-sectional study comparing patients on PF timolol maleate (48 eyes) with healthy controls (40 eyes) found that TBUT was significantly higher in controls compared to patients on timolol maleate-PF [26].

In an animal study involving New Zealand white rabbits, Russ et al. found that timolol increases subepithelial collagen density and extracellular matrix (ECM) more than prostaglandin analogs (PGAs), thus potentially interfering with glaucoma filtration surgery outcomes [27].

#### 3.1.2. Prostaglandin Analogs

PGAs decrease IOP by remodeling the ECM in the ciliary muscle bundles, iris root, and sclera, thereby increasing uveoscleral outflow. In addition, there may be remodeling of corneal collagen fibers, resulting in decreased central corneal thickness. Other well-documented PGA side effects include skin pigmentation, MGD, conjunctival hyperemia, pseudo-dendritic keratitis, periorbitopathy, eyelid pigmentation, and hypertrichosis [5,28].

In 2016, Yamada et al., using human non-pigmented ciliary epithelial cells, studied bimatoprost, latanoprost, and tafluprost and found elevated matrix metalloproteinase (MMP) levels and reduced levels of tissue inhibitors metalloproteinases (TIMP-1 and TIMP-2) [29].

Similarly, a Turkish study in 2016 involving 70 glaucoma patients found that long-term use of PGAs was significantly associated with a higher prevalence of MGD (92% vs. 58.3% in non-PGA users). These patients also exhibited worse OSDI scores (22.5 ± 24.3 vs. 1.9 ± 3.4), tear film stability, and MGD (95.7%) [30].

#### 3.1.3. Alpha-Adrenergic Agonists

Alpha-adrenergic agonists (brimonidine and apraclonidine) are selective sympathetic agonists of the α2 receptor and thus have multiple effects: (1) decreased AH production, (2) increased uveoscleral outflow, and (3) increased trabecular meshwork (TM) outflow.

Research has shown that the common follicular conjunctivitis may result from alpha-adrenergic agonists’ effect on reducing the volume of conjunctival cells, thereby widening intracellular spaces and permitting potential allergens to penetrate subepithelial tissue [31]. The incidence of brimonidine allergy ranges from 4.7% to 25%, with the average time from the start of treatment to the onset of allergic follicular conjunctivitis being six to nine months. However, this interval can vary widely, from as short as 14 days to as long as 12 months and is independent of the presence of BAK [31].

These agents should not be used in children due to the potential for central nervous system depression given that topical alpha-adrenergic agonists are not weight-adjusted [32].

#### 3.1.4. Carbonic Anhydrase Inhibitors

Carbonic anhydrase inhibitors (CAIs) can adversely affect tear film stability, with surface conditions such as hyperemia, blepharitis, dry eyes, and tearing occurring in less than 3% of cases [33,34]. Terai and colleagues discovered that brinzolamide reduced basal tear secretion, although it did not significantly affect TBUT. Specifically, dorzolamide was found to reduce basal tear secretion by 14.3% at 60 min and by 17.3% at 90 min post-application [34]. CAIs are generally avoided in patients who have sulfa allergies or a history of nephrolithiasis.

#### 3.1.5. Cholinergic Agonists

Cholinergic agonists (pilocarpine and carbachol) activate the muscarinic type 3 receptors on ciliary smooth muscle cells, resulting in expansion of the juxtacanalicular portion of the TM and expansion of the Schlemm’s canal [35]. It also acts on the iris sphincter muscles, inducing miosis. An in vitro study using immortalized human meibomian gland epithelial cells (IHMGEC) found that pilocarpine led to a dose-dependent decrease in IHMGEC proliferation, leading to cell atrophy and death [36]. Adverse effects of pilocarpine include conjunctival hyperemia, MGD, blepharitis, pseudo-pemphigoid cicatrizing conjunctivitis, burning/stinging, eye pain, blurred vision, increased corneal staining, and headache [5,37].

#### 3.1.6. Latanoprostene Bunod

Latanoprostene bunod (LBN) 0.024%, commercially available as Vyzulta^®^, is a nitric oxide (NO)-donating prostaglandin F2α analogue which increases the aqueous outflow both by uveoscleral and trabecular pathways. The NO relaxes TM cells and facilitates the trabecular outflow. NO also may regulate ocular blood flow and may promote retinal ganglion cell (RGC) survival in the eye. The latanoprost acid, the second active metabolite, shares the familiar mechanism of action of PGAs by increasing the uveoscleral outflow. The most common ocular adverse effects of LBN were conjunctival hyperemia, hypertrichosis, eye irritation, eye pain, and an increase in iris pigmentation [38].

#### 3.1.7. Netarsudil

Netarsudil 0.02% (Rhopressa^®^) is a rho-associated kinase (ROCK) inhibitor and a norepinephrine transporter (NET) inhibitor. It has a tri-faceted mechanism of action: it increases the TM outflow, decreases episcleral venous pressure, and decreases AH production [39]. Furthermore, it may decrease RGC loss by improving optic nerve head perfusion by its effect on endothelin 1. Common side effects include conjunctival hyperemia, subconjunctival bleeding, SPK, corneal edema, and whorl or honeycomb keratopathy [38].

### 3.2. Preservatives

Preservatives in glaucoma medications are crucial for preventing microbial contamination and ensuring their longevity, safety, and efficacy. These preservatives can be broadly categorized as detergents, oxidative agents, and ionic tamponade agents (Table 4).

#### 3.2.1. Detergents

Detergents act by disrupting the cell membranes of microbials, thus preventing contamination. BAK and Polyquad^®^ (polidronium chloride) are among the most well-known in this category. BAK, a cationic detergent, is commonly used in approximately 70% of multi-dose glaucoma drops at concentrations ranging from 0.003% to 0.02% [52]. BAK is highly cytotoxic to the corneal and conjunctival epithelial cells, including the limbal stem cells. Its mode of action is by damaging the deoxyribonucleic acid (DNA), disrupting tight junctions, and inducing cell death through apoptosis or necrosis [53]. Although its lipophilic nature allows easier penetration of topical drugs through the corneal epithelium, it may also cause ocular surface irritation and inflammation [54]. Polyquad^®^, a quaternary ammonium compound, has a polymeric structure that limits its penetration into cell membranes, making it less cytotoxic compared to BAK.

Several researchers found wide variation in the prevalence of OSD among patients using topical glaucoma medications (37–91%) [55]. Ramli et al. found higher rates of corneal staining (63% vs. 36%), abnormal Schirmer’s tests (39% vs. 25%), and moderate OSDI symptoms (17% vs. 7%) in patients using BAK-containing medications compared to the control group [55]. They also found a strong association between the number of eye drops, the presence of preservatives, and the severity of OSD.

Another multicenter cross-sectional study in 9658 patients with open-angle glaucoma assessed the prevalence of toxicity when using beta-blocker eye drops with or without preservatives [48]. The researchers found that patients using P drops reported significantly more symptoms, such as pain during instillation (48% vs. 19%), foreign body sensation (42% vs. 15%), stinging or burning (48% vs. 20%), and dry eye sensation (35% vs. 16%), compared to those on PF drops. When patients were switched from P to PF drops, there was a significant reduction in ocular symptoms and signs, highlighting the benefits of PF formulations.

Chronic use of topical anti-glaucoma medications may interfere with wound healing after glaucoma filtering procedures [56]. Histological specimens from patients undergoing filtering surgery have shown reductions in goblet cells and increased inflammatory cells, such as macrophages, lymphocytes, fibroblasts, and mast cells.

In comparison, Polyquad^®^ (polyquaternium-1)-containing drops have fewer adverse effects than BAK. For instance, OSDI scores were significantly lower in patients using Polyquad^®^-preserved travoprost compared to BAK-preserved travoprost [57]. Additionally, in vitro studies with human TM cells showed higher cell viability with Polyquad^®^-preserved formulations versus BAK-preserved ones [58]. Interestingly, compared to Polyquad^®^, BAK has been shown to be associated with dose-dependent reductions in TM cell viability and increased levels of MMP-9, a factor in glaucoma pathogenesis [58].

#### 3.2.2. Oxidative Agents

The most common oxidative agent used in ocular pharmacology is stabilized oxychloro complex (SOC, Purite^®^). It disrupts microbial protein synthesis through the production of chlorine dioxide. SOC is most suitable for chronic use because of its unique ability to break down into components already found in the tears (Na^+^, Cl^−^, O_2_, and H_2_O). This property enhances its tolerability, reduces toxicity, and improves patient compliance [51].

In a 12-month, randomized, multicenter, double-masked study, brimonidine-Purite^®^ 0.15% and 0.2% were compared to brimonidine-BAK 0.2% in patients with glaucoma or ocular hypertension. The results showed that brimonidine-Purite^®^ 0.15% provided comparable IOP reduction to brimonidine 0.2% with significantly lower incidence of allergic conjunctivitis and hyperemia, higher patient satisfaction, and comfort ratings [59].

#### 3.2.3. Ionic Tamponade Agents

These agents, such as SofZia^®^, are buffers that maintain the pH and osmolarity of the solution and enhance its comfort and stability. SofZia^®^ contains borate, sorbitol, propylene glycol, and zinc and has both antibacterial and antifungal properties. It degrades quickly upon contact with cations on the ocular surface, resulting in less cytotoxicity compared to BAK [60].

Kanamoto’s group, in 2015, studied the ocular surface tolerability of tafluprost with 0.001% BAK versus travoprost preserved with SofZia^®^ in 195 patients with glaucoma. They found that SPK and conjunctival hyperemia scores were lower in the tafluprost group compared to the travoprost group (*p* = 0.038) [61].

### 3.3. Penetration Enhancers

Penetration enhancers are used in topical ocular medications to enable active ingredients to penetrate the ocular surface through the transcellular or paracellular routes. One of the most common penetration enhancers used in anti-glaucoma medications is BAK. Other examples include chelating agents, cyclodextrins, crown ethers, bile acids, salts, cell-penetrating peptides, saponin, ethylenediaminetetraacetic acid (EDTA), paraben, and Transcutol^®^. Each penetration enhancer, however, has its own specific side effects [62].

## 4. Discussion

### 4.1. Management of OSD Caused by Glaucoma Medications

Managing glaucoma patients with OSD requires a multi-faceted approach focused on reducing ocular surface toxicity, improving tear film stability, and controlling inflammation. Switching to PF medications, using supportive treatments for the ocular surface, and regular monitoring are key components of this strategy. In addition, advanced therapies and surgical options can be considered for patients with severe or refractory OSD.

#### 4.1.1. Step 1: Modify Glaucoma Therapy

Transitioning to a PF version of the identical medication enhances OSD outcomes while maintaining the same hypotensive effect. A 2010 study from Finland found that replacing a P prostaglandin analog with a PF variant resulted in a significant reduction in OSD symptoms such as itching (46.8% to 26.5%), irritation/burning/stinging (56.3% to 28.4%), dry eye sensation (64.6% to 39.4%), abnormal fluorescein staining of the cornea (81.6% to 40.6%), conjunctival hyperemia (84.2% to 60%). Furthermore, TBUT increased from 4.5 ± 2.5 to 7.8 ± 4.9 s [63].

Similarly, an Italian study also found that switching BAK-containing beta-blocker formulations to PF versions resulted in a notable reduction of OSD symptoms, specifically, burning and stinging (40% to 20%), foreign body sensation (31% to 14%), dryness sensation (23% to 14%), and tearing (21% to 14%) [64].

Similar findings have also been reported even for combined PF brimonidine tartrate medications and resulted in improved patient comfort, satisfaction, and adherence to the treatment. When PF options are not available, one may try formulations containing Polyquad^®^ or SofZia^®^ [65].

#### 4.1.2. Step 2: Ocular Surface Lubrication, Anti-Inflammatory Treatment, and Other Supplemental Therapies

The Dry Eye Workshop (DEWS) II Subcommittee’s recommendations have simplified diagnosing DED. The diagnosis can be made if a patient exhibits a NITBUT (less than 10 s), high tear osmolarity (>308 mOsm/L), ocular surface staining (more than five spots on the cornea), accompanied by symptomatic evaluation using validated scoring systems like the OSDI [66]. In the early stages of OSD, the elimination of P medications combined with the use of PF artificial tears may be sufficient [67]. Proper eyelid hygiene with a frequent cleansing routine and warm compresses may help alleviate associated blepharitis.

##### Anti-Inflammatory Treatment (Cyclosporine A and Topical Steroids)

In addition, clinicians may consider starting anti-inflammatory treatment using topical steroids or cyclosporine A (CsA) drops. A 2023 South Korean randomized clinical trial demonstrated that 0.05% topical CsA significantly improved OSD parameters, increased Schirmer’s test scores, TBUT, and TMH, and decreased ocular staining and MMP-9 positivity in treated eyes [68].

In some patients not responding to conventional treatment, the use of topical steroids may be essential, though, physicians must be aware of their role in elevating IOP and inducing cataracts. Carbon-20 ester steroids (loteprednol) are often preferred over carbon-20 ketone steroids (prednisolone, dexamethasone, and fluorometholone) [69].

##### Omega-3 Fatty Acid Supplementation

According to a 2019 meta-analysis consisting of 17 randomized clinical trials, omega-3 fatty acid supplementation has been associated with decreased dry eye symptoms and corneal staining along with increased TBUT and Schirmer’s test values [70].

##### Vitamin A Eye Gel

Vitamin A may offer a promising therapeutic option for individuals with dry eye syndrome. The use of vitamin A palmitate as an eye gel has demonstrated beneficial effects on the morphology of the conjunctival epithelium and density of goblet cells in patients undergoing long-term treatment with topical PGAs [71].

##### Autologous Serum Eye Drops

Autologous serum eye drops, prepared by centrifuging a patient’s own blood to separate the liquid and cellular components, may be used for moderate to severe OSD. Studies have shown that autologous serum eye drops contain cytokines and biochemical factors that are important for ocular surface health, including epithelial growth factor, TGF-β, and fibronectin [72].

##### Cryopreserved Amniotic Membranes

For patients with refractory OSD, a self-retained sutureless cryopreserved amniotic membrane (cAM) with a poly-carbonate ring frame can be placed under topical anesthetic in the clinic for an average duration of five days. cAM exhibits anti-inflammatory properties and promotes corneal healing through mechanical protection of the epithelial surface. In a multi-center, retrospective study involving 89 eyes from 77 patients with moderate-to-severe OSD, placement of a cAM for two days improved DEWS scores, corneal staining, visual symptoms, and ocular discomfort at one-week, one-month, and three-month follow-up [73]. Reported side effects include foreign body sensation and temporary blurred vision.

#### 4.1.3. Step 3: Surgical Treatment

Regarding patients who are intolerant to topical medications and show no improvement with PF medications or oral CAIs, surgical procedures may be considered, such as selective laser trabeculoplasty, trabeculectomy, glaucoma drainage devices (GDDs), and minimally invasive glaucoma surgery (MIGS) [74]. However, each surgical intervention is fraught with its own complications and must be considered carefully.

### 4.2. Future Directions in the Management of Ocular Surface Diseases

There are exciting new therapies and technologies in the pipeline for managing OSD in patients using topical glaucoma medications. This study could be improved further by conducting a detailed systematic literature review regarding the efficacy of these novel treatments. A brief overview of some of these innovative therapies is described below, including sustained-release drug delivery systems (extraocular and intraocular) [75], intense pulsed light therapy, thermal pulsation devices, photobiomodulation, nanoparticles, gene alteration, stem cell applications, umbilical cord blood serum eye drops, and acupuncture (Table 5).

#### 4.2.1. Sustained-Release Drug Delivery Systems

Sustained-release systems are broadly categorized into extraocular or intraocular delivery platforms that offer a consistent drug concentration at the target site over a longer duration [75]. They offer promising alternatives to current challenges of ocular surface toxicity, inadequate IOP control, and non-compliance.

##### Extraocular Drug Delivery Platforms

Extraocular systems include gel-forming eye drops, ocular inserts, contact lenses, and punctal plugs [75]. Gel-forming formulations, such as SoliDrop^®^ (Otero Therapeutics, University of Pittsburgh, Pittsburgh, PA, USA) transform into a semi-solid gel upon contact with tears, extending drug residence time [76].

Ocular inserts, such as Bimatoprost Ocular Ring^®^ (AbbVie, Chicago, IL, USA) and Topical Ophthalmic Drug Delivery Device^®^ (TODDD) (Amorphex Therapeutics, Andover, MA, USA), are placed in the conjunctival fornix, releasing the drug through diffusion and bioerosion [77,78].

Contact lenses are well-tolerated and are used in various forms to deliver ocular medications with the added benefits of minimal interference with vision and prolonged drug residence time. For example, contact lenses impregnated with timolol-vitamin E complex and Methafilcon lenses loaded with latanoprost utilize passive diffusion [79,80]. Another contact lens option uses molecular imprinting during the fabrication and polymerization process to create drug receptor sites, enhancing drug retention and release [81].

Punctal plugs, such as Evolute^®^ (Mati Therapeutics, Austin, TX, USA) and OTX-TP^®^ (Ocular Therapeutix, Bedford, MA, USA), are inserted in the lid puncta, delivering travoprost through diffusion while maintaining tear film integrity [82,83].

##### Intraocular Drug Delivery Systems

Intraocular drug delivery devices are designed to be inserted in the eye for prolonged drug release. They, however, require surgical intervention and may carry risks, such as damage to the eye, hypotony, IOP spikes, retinal detachment, and endophthalmitis [75].

Among the intracameral implants, DURYSTA^®^ (AbbVie) is an FDA-approved biodegradable bimatoprost implant that lowers IOP for 4–6 months [84]. Similarly, ENV515^®^ (Envisia Therapeutics, Durham, NC, USA) and OTX-TIC^®^ (Ocular Therapeutix) release travoprost over a similar period [85,86]. Glaukos’ product iDose Travoprost^®^ (Aliso Viejo, CA, USA) is inserted within the TM, offering a year-long IOP control, but requires a more prolonged and invasive procedure [87].

Another alternative, such as subconjunctival implant Eye-D VS-101^®^ (Biolight Life Sciences, Tel Aviv, Israel), offers a less invasive option and ease of injection, and still provides sustained release of latanoprost over several months [78,88].

#### 4.2.2. Innovative Technological Devices

New technological innovations in OSD management include intense pulsed light (IPL) therapy, thermal pulsation devices, and photobiomodulation.

##### Intense Pulsed Light Therapy

During IPL treatments (OptiLight^®^, Lumenis, Yokneam, Isreal), protective eyepieces cover the eyes and high intensity light pulses are directed above the eyebrows, lower eyelids, zygomatic region, and nose, leading to destruction of abnormal blood vessels while maintaining meibomian gland architecture and function [89]. IPL therapy consists of four 20 minute sessions at three-week intervals followed by maintenance therapy every three to six months. A 2022 meta-analysis of 15 randomized controlled clinical trials found that compared to controls, patients who received IPL treatments had improved OSDI scores, standard patient evaluation of eye dryness (SPEED) scores, artificial tear usage, tear film lipid layer, meibomian gland quality, meibomian gland expression, corneal fluorescein staining, TBUT, and NITBUT [90].

##### Thermal Pulsation Devices

Thermal pulsation devices such as LipiFlow^®^ (Johnson & Johnson Vision, Jacksonville, FL, USA) consist of disposable eyepieces with attached lenses that protect the cornea while direct heat and pressure are applied over the eyelids to liquefy and express meibomian gland secretions. Although IPL and thermal pulsation devices are FDA-approved, they have not yet solidified their role in daily clinical practice. A 2022 meta-analysis found that compared to controls, patients who received LipiFlow^®^ treatments had improvements in OSDI scores, SPEED scores, and meibomian glands yielding secretion scores [91].

##### Photobiomodulation

Photobiomodulation, also known as low-level light therapy, uses a mask that covers the face and eyelids and emits light in the red (633 nm) or blue (428 nm) wavelength for 15–30 min. Blue light has been shown to inhibit microbial growth while red light generates heat, promotes tissue repair, and decreases inflammation. A US prospective pilot study with 30 patients who received three 15 minute sessions at one-week intervals of photobiomodulation with red light found a statistically significant improvement in NITBUT, TMH, tear film lipid layer thickness, and Schirmer’s test [92]. A triple-masked, randomized controlled trial, Photobiomodulation With REd vs. BluE Light (REBEL), is currently being conducted at Aston University, United Kingdom [93].

#### 4.2.3. Other Emerging Therapies

A number of emerging therapies that have shown promise in improving aqueous outflow and neuroprotection include nanoparticles, gene therapy, and stem cell applications [94]. Umbilical cord blood serum eye drops and acupuncture may improve OSD as well.

##### Nanoparticles

Nanoparticles consisting of certain polymers, lipids, or metals may improve drug bioavailability, enabling slow release while reducing adverse effects. In a lab study using New Zealand white rabbits, timolol-loaded gold nanoparticles embedded in contact lenses led to increased timolol concentrations in tear fluid, conjunctiva, and iris-ciliary muscles [95,96].

##### Gene Therapy

Preclinical ocular gene therapy alters gene expression via nanoparticles or viral vectors. One arm targets the TM to increase AH outflow. A US study with 30 Brown Norway rats utilized recombinant adeno-associated virus (AAV) vector-mediated gene therapy to target de novo prostaglandin F2α synthesis in the AC and found a reduction in IOP over 12 months [97].

The second arm of gene research focuses on neuroprotection by targeting RGC cell loss by increasing the expression of neurotrophins, such as brain-derived neurotrophic factor (BDNF) and ciliary neurotrophic factor (CNTF), antioxidant genes, anti-inflammatory genes, cell cycle regulators, and protease inhibitors. To prove this hypothesis, Japanese investigators used optic nerve crush (ONC) glaucoma mouse models and injected them with intravitreal injections of AAV-F-iTrkB (AAV farnesylation of the intracellular domain of TrkB) and found increased axon regeneration [98].

##### Stem Cell Applications

Stem cells can be used to improve TM structure and function, promote RGC survival, and improve corneal barrier dysfunction [94]. An animal study using a Long-Evans rat model of ocular hypertension found that when bone-marrow derived mesenchymal stem cells (MSCs) were tagged and injected into the AC, there was a significant decrease in IOP and MSCs were located in the ciliary processes and TM [99]. In a study conducted at the University of Pennsylvania, human-induced pluripotent stem cells (hiPSCs) were differentiated to mature RGCs in vitro and then injected intravitreally into mice, and it was found that hiPSCs integrated into the RGC layer for a successful transplantation rate of 94% at five-months follow up [100]. In a 2023 Japanese study, a conditioned-medium containing factors secreted from human adipose-derived MSCs was shown to decrease BAK-induced inflammation of human corneal epithelial cells in an in vitro model [101]. Next, the researchers, using a DED rat model, found decreased corneal fluorescein staining and improved tear production [101].

##### Umbilical Cord Blood Serum Eye Drops

Umbilical cord blood serum (CBS), readily available from blood banks, can be used as eye drops. These drops contain high levels of growth factors (epithelial growth factor and TGF-β1) and anti-inflammatory cytokines. In an observational, longitudinal, interventional study conducted in Singapore, 40 patients with refractory OSD were started on CBS eye drops. On average, the patients used the CBS drops 2.23 times per day with an average of 5.5-months follow-up and were found to show improvement in kerato-epitheliopathy staining score, TBUT, and SPEED score [102].

##### Acupuncture

Acupuncture may be beneficial in OSD based on its ability to downregulate proinflammatory cytokines and increase the release of acetylcholine in the lacrimal glands promoting tear secretion. A 2022 meta-analysis with 394 patients who underwent acupuncture showed significant improvement in OSDI scores and Schirmer’s test scores, including TBUT, compared to controls [103].

## 5. Conclusions

Clinicians must be aware of the close association and high prevalence between OSD and long-term glaucoma therapy. If left unchecked, OSD may affect quality of life and treatment adherence, thus negatively impacting glaucoma care. Initially, transitioning to PF glaucoma medications is a crucial step, and combining with CsA or topical steroids may be beneficial. For patients with refractory OSD or uncontrolled IOP, surgical interventions may offer some benefits. Overall, a comprehensive and multifaceted management approach is essential to optimize both ocular surface health and effective glaucoma treatment.

## Figures and Tables

**Table 1:** PICOS criteria for inclusion of studies.

Parameter	Description
Population	Patients with glaucoma regardless of study location
Intervention	Focusing on patients using anti-glaucoma eye drops with or without preservatives
Comparison	Patients using topical eye drops with or without preservatives
Outcomes	OSDI, Schirmer’s test, corneal and conjunctival staining (fluorescein, lissamine green), conjunctival hyperemia, meibography, TMH, TBUT, NITBUT
Study Design	Cohort, cross-sectional, case-control, randomized or nonrandomized controlled (or uncontrolled) trials, or reviews

**Table 2:** Characteristics of glaucoma medications.

Medications	Mechanism of Action	Dosing & Concentrations	OSD or Other Complications	IOP Reduction
Beta-adrenergic blockers (timolol, levobunolol, betaxolol, metipranolol) [5,20,21,22,23,24,25,26,27]	Decrease aqueous humor (AH) production via blockade of beta-adrenergic receptors on the ciliary epithelium	Once or twice daily; 0.25–0.5%	Conjunctival goblet cell loss, MGD, SPK, and pseudo-pemphigoid cicatrizing conjunctivitis	~20–30%
Prostaglandin analogues (latanoprost, bimatoprost, travoprost, tafluprost) [5,28,29,30]	Increase uveoscleral outflow by remodeling the ECM and regulating matrix metalloproteinases	Once daily; 0.0015–0.03%	MGD, skin pigmentation, conjunctival hyperemia, pseudo-dendritic keratitis, periorbitopathy, eyelid pigmentation, and hypertrichosis	~25–35%
Alpha-adrenergic agonists (brimonidine, apraclonidine) [31,32]	Selective sympathetic agonists (α2); decrease AH production, and increase uveoscleral and trabecular meshwork (TM) outflow	2–3 times daily; 0.1–0.5%	Allergic follicular conjunctivitis, contact dermatitis, blepharitis, and systemic hypotension	up to 26%
Carbonic anhydrase inhibitors (dorzolamide, brinzolamide), (oral: acetazolamide, methazolamide) [33,34]	Decrease AH production by inhibiting carbonic anhydrase enzyme in the ciliary processes	2–4 times daily; 1–2%	Ocular surface irritation, reduction of basal tear secretion, and blepharitis	~15–20%
Cholinergic agonists (pilocarpine, carbachol) [5,35,36,37]	Muscarinic receptor agonists; increase TM outflow	4 times daily; 1–4%	MGD, blepharitis, pseudo-pemphigoid cicatrizing conjunctivitis, blurred vision, myopia, miosis, iris cysts, and retinal detachment	~15–25%
Latanoprostene bunod (Vyzulta^®^) [38]	Induces TM expansion and vasodilation of episcleral veins, thereby increasing AH outflow	Once daily; 0.024%	Hyperemia, hypertrichosis, and eye irritation	~35%
Rho Kinase inhibitors (netarsudil—Rhopressa^®^) [38,39]	Decrease episcleral venous pressure, increase TM outflow, and decrease AH production via inhibition of rho kinase enzyme	Once daily; 0.02%	Conjunctival hyperemia and hemorrhage, corneal edema, and SPK	~25–30%
Dorzolamide and timolol maleate solution (combined)	Decrease AH production via a combination of carbonic anhydrase and beta-adrenergic receptor blockade	Twice daily; timolol 0.5%, dorzolamide 2%	Conjunctival goblet cell loss, MGD, SPK, pseudo-pemphigoid cicatrizing conjunctivitis, ocular surface irritation, reduction of basal tear secretion, and blepharitis	~30–35%
Brimonidine tartrate and timolol maleate solution (combined)	Decrease AH production, increase uveoscleral outflow, and increase TM outflow via a combination of alpha and beta-adrenergic receptor blockade	Twice daily; timolol 0.5%, brimonidine 0.2%	Allergic follicular conjunctivitis, contact dermatitis, blepharitis, conjunctival goblet cell loss, MGD, SPK, and pseudo-pemphigoid cicatrizing conjunctivitis	~30–35%
Netarsudil and latanoprost solution (Rocklatan^®^)	Decrease episcleral venous pressure, increase TM outflow, and decrease AH production via a combination of rho kinase inhibition and prostanoid receptor induction	Once daily; netarsudil 0.02%, latanoprost 0.005%	Hyperemia, conjunctival hemorrhage, MGD, lid pigmentation, pseudo-dendritic keratitis, periorbitopathy, and hypertrichosis	~30–36%
Brimonidine and brinzolamide solution (combined)	Decrease AH production, and increase uveoscleral and TM outflow via inhibition of carbonic anhydrase and alpha-adrenergic receptors	3 times daily; brimonidine 1%, brinzolamide 0.2%	Ocular surface irritation, reduction of basal tear secretion, blepharitis, allergic follicular conjunctivitis, and contact dermatitis	~21–35%

Legend: OSD = ocular surface disease, IOP = intraocular pressure, AH = aqueous humor, MGD = meibomian gland dysfunction, SPK = superficial punctate keratitis, ECM = extracellular matrix, TM = trabecular meshwork.

**Table 3:** Key studies regarding ocular surface disease in patients on glaucoma medications.

Glaucoma Agents and Patient Characteristics	Study Methods	Study Results	Authors, Country, and Year
Newly diagnosed treatment-naïve POAG patients vs. those on topical anti-glaucoma medications	A prospective cohort study conducted on 120 eyes with POAG (60 on topical anti-glaucoma drops and 60 treatment-naïve eyes).	At 3, 6, and 12 months, the OSDI score, TBUT, Schirmer’s test, TMH, and TMD had significantly better values in the treatment-naïve group in comparison to the medicated group (*p* < 0.0001).	Srivastava et al. India, 2024 [40]
Patients with open-angle glaucoma or OHT on topical anti-glaucoma medications vs. healthy subjects	In this cross-sectional study, 75 patients were using topical anti-glaucoma medications and 65 were treatment-naïve subjects. OSDI, Schirmer’s test, TBUT, fluorescein staining, and CET were evaluated.	The treatment group had a significantly shorter TBUT, shorter Schirmer’s test, and greater fluorescein staining than those of the control group (*p* < 0.05). The mean CET of patients with glaucoma was significantly lower than that of controls in the central, paracentral, mid-peripheral, and peripheral zones (50.6 vs. 53.1 µm; *p* < 0.001). The number of medications and duration of treatment also affected the CET in all zones (*p* < 0.05).	Ye et al. China, 2022 [41]
Glaucoma patients on topical anti-glaucoma medications vs. healthy controls	94 patients with glaucoma on topical medications (study group) and 94 patients in the treatment-naïve control group were assessed using OSDI, TBUT, lissamine green staining, and Schirmer’s test.	OSDI scores were significantly higher in the study group (72.4%) vs. controls (44.6%). Similarly, the study group had decreased tear production (84% vs. 53%, respectively), abnormal TBUT (67.1% vs. 47.8%), and positive lissamine green staining (36.2% vs. 31.8%) compared to the control group.	Pai and Reddy India, 2018 [42]
Patients with POAG or OHT on topical anti-glaucoma medications vs. healthy controls	211 eyes of patients with POAG or OHT on topical medication were recruited. Controls consisted of 51 eyes. Outcome measures were fluorescein corneal staining score, TMH, TBUT, and OSDI.	Compared to controls, significantly higher OSDI (10.24 vs. 2.5; *p* < 0.001) and corneal staining (≥1: 64.93% vs. 32.61%; *p* < 0.001) scores were recorded in the medication group. No significant differences in TBUT and TMH were observed between groups.	Pérez-Bartolomé et al. Spain, 2017 [43]
Glaucoma patients on topical anti-glaucoma medications vs. OHT patients or relatives of glaucoma patients not on topical medications	In this cross-sectional study, 109 participants (79 on topical medications and 30 controls) were evaluated via OSDI, Schirmer’s test, TBUT, and fluorescein staining.	The medication group had significantly shorter TBUT (6.0 vs. 9.5 s; *p* < 0.03), greater fluorescein staining (1.0 vs. 0; *p* < 0.001), and higher impression cytology grade than the control group (1.0 vs. 0.6; *p* < 0.001).	Cvenkel et al. Slovenia, 2015 [44]
Patients with POAG on topical anti-glaucoma medications vs. healthy controls	Age-matched patients were assigned to 2 groups: the glaucoma group (31 patients) and the treatment-naïve control group (30 patients). Each patient was assessed with OSDI, conjunctival/corneal staining, and TBUT.	OSDI scores of the glaucoma group positively correlated to the amount and duration of drops used. The glaucoma group had a higher mean OSDI score than the control group (18.97 vs. 6.25). Abnormal TBUT and staining scores were seen in the glaucoma group compared with the control group (68% vs. 17%).	Saade et al. USA, 2015 [45]
Patients with glaucoma or OHT on 0, 1, or ≥2 topical anti-glaucoma medications	39 patients treated for glaucoma or OHT and 9 untreated patients were included in this study. Corneal sensitivity was measured using the Cochet-Bonnet esthesiometer, Schirmer’s test, TBUT, corneal and conjunctival fluorescein staining, and OSDI.	Corneal sensitivity of patients treated with IOP-lowering medications was negatively correlated to the number of instillations of P drops (*p* < 0.001) and duration of treatment (*p* = 0.001). There was no significant difference in OSDI or Schirmer’s test scores between the groups.	Van Went et al. France, 2011 [46]
Patients with POAG, pseudoexfoliation glaucoma, pigment dispersion glaucoma, or OHT on topical anti-glaucoma medications	This prospective observational study assessed OSDI in 630 patients with POAG, pseudoexfoliation glaucoma, pigment dispersion glaucoma, or OHT who were on topical IOP-lowering medications.	305 patients (48.4%) had an OSDI score indicating either mild, moderate, or severe OSD symptoms. Higher OSDI scores were observed in patients using multiple IOP-lowering medications (*p* = 0.0001).	Fechtner et al. USA, 2010 [47]
Patients using P vs. PF topical beta-blocker drops	In a multicenter cross-sectional survey in four European countries, ophthalmologists in private practice enrolled 9658 patients using P or PF beta-blocking eyedrops between 1997 and 2003. Subjective symptoms, conjunctival and palpebral signs, and SPK were assessed before and after a change in therapy.	Palpebral, conjunctival, and corneal signs were significantly more frequent (*p* < 0.0001) in the P-group than in the PF-group, such as pain or discomfort during instillation (48% vs. 19%), foreign body sensation (42% vs. 15%), stinging or burning (48% vs. 20%), and dry eye sensation (35% vs. 16%). A significant decrease (*p* < 0.0001) in all ocular symptoms was observed in patients who switched from P to PF eye drops.	Jaenen et al. Belgium, 2007 [48]
Patients with POAG or OHT using P vs. PF topical anti-glaucoma medications	This prospective epidemiological survey was carried out in 1999 by 249 ophthalmologists on 4107 patients. Ocular symptoms, conjunctiva, and cornea were assessed between P and PF eye drops.	All symptoms were more prevalent with P than with PF drops (*p* < 0.001): discomfort upon instillation (43% vs. 17%), burning-stinging (40% vs. 22%), foreign body sensation (31% vs. 14%), dry eye sensation (23% vs. 14%), and tearing (21% vs. 14%). An increased incidence (>2 times) and duration of ocular signs were seen with P eye drops, which decreased upon switching to PF drops (*p* < 0.001).	Pisella et al. France, 2002 [49]

Legend: POAG = primary open-angle glaucoma, OSDI = ocular surface disease index, TBUT = tear break-up time, TMH = tear meniscus height, TMD = tear meniscus depth, OHT = ocular hypertension, CET = corneal epithelial thickness, IOP = intraocular pressure, P = preserved, PF = preservative-free, SPK = superficial punctate keratitis.

**Table 4:** Common preservatives in ocular formulations [51].

Category	Examples
Detergents	benzalkonium chloride (BAK) polidronium chloride (polyquaternium-1, Polyquad^®^)
Oxidative agents	stabilized oxychloro complex (SOC, Purite^®^) sodium perborate (GenAqua^®^)
Ionic tamponade agents	borate, sorbitol, propylene glycol, and zinc (SofZia^®^)

**Table 5:** Future directions in the management of ocular surface disease in glaucoma.

Product	Product Status	Mechanism of Action
Extraocular Drug Delivery Systems		
Gel-forming drops A. SoliDrop^®^ gel solution (Otero Therapeutics) [76]	Preclinical	The higher viscosity gel-containing drops stay on the surface of the eyes for a longer period of time, thereby providing greater surface protection.
Ocular inserts Bimatoprost Ocular Ring^®^ (AbbVie) [77] Topical Ophthalmic Drug Delivery Device^®^ (TODDD^®^, Amorphex Therapeutics) [78]	Bimatoprost Ocular Ring^®^ is in Phase 2, and TODDD^®^ is in Phase 1.	Ocular rings containing anti-glaucoma medications may be inserted in the upper and lower fornices for slow release, thickening the precorneal tear film and protecting the eye.
Passive Diffusion Contact Lenses (PDCLs) Vitamin-E CLs loaded with timolol (University of Florida, USA) [79] Methafilcon lenses loaded with latanoprost (Harvard Medical School, USA) [80]	Preclinical	Anti-glaucoma drug impregnated CLs release active ingredients through passive diffusion.
Molecular Imprinted Contact Lenses (MICLs) A. Timolol maleate loaded MICL (University of Kerala, India) [81]	Preclinical	During the fabrication of MICLs, molecular sites akin to drug receptor sites are embedded in the polymer, increasing loading and sustained release of anti-glaucoma drugs.
Punctal Plugs (PPs) Evolute^®^ (travoprost-loaded, Mati Therapeutics) [82] OTX-TP^®^ (travoprost-loaded, Ocular Therapeutix) [83]	Evolute^®^ is in Phase 2, and OTX-TP^®^ is in Phase 3.	PPs block tear drainage and increase tear film contact time with the ocular surface.
Intraocular Drug Delivery Systems		
Anterior Chamber (AC) Intracameral Implants (II) DURYSTA^®^ (bimatoprost, AbbVie) [84] ENV515^®^ (travoprost, Envisia Therapeutics) [85] OTX-TIC^®^ (travoprost, Ocular Therapeutix) [86] iDose^®^ (travoprost, Glaukos Corporation) [87]	Phase 2 or 3	II are injected in the AC or anchored in the trabecular meshwork (TM) and slowly release medications over months. They are either biodegradable hydrogel or titanium implants.
Subconjunctival Implants (SI) A. Eye-D VS-101^®^ (latanoprost, Biolight Life Sciences) [78,88]	Phase 1 or 2a	SI impregnated with glaucoma drugs are injected subconjunctivally to provide slow drug release.
Innovative Technological Devices		
Intense Pulsed Light (IPL) Therapy A. OptiLight^®^ (Lumenis) [89,90]	Phase 4	High intensity light pulses are directed around the eyes, which may destroy abnormal blood vessels and alter meibomian gland architecture and function.
Thermal Pulsation Devices (TPD) A. LipiFlow^®^ (Johnson & Johnson Vision) [91]	Phase 4	TPDs consist of disposable eyepieces which direct heat and pressure over the eyelids to liquefy and express meibomian gland secretions.
Photobiomodulation Low-level light therapy with near-infrared light-emitting diodes (Dankook University, South Korea) [92] Photobiomodulation With REd vs. BluE Light (REBEL) Study (Aston University, United Kingdom) [93]	Phase 2	Photobiomodulation uses a mask to emit light over the face and eyelids. Blue light inhibits microbial growth while red light generates heat, promotes tissue repair, and decreases inflammation.
Other Emerging Therapies		
Nanoparticles A. Timolol-loaded gold nanoparticles (Uka Tarsadia University, India) [94,95,96]	Preclinical	Nanoparticles consisting of certain polymers, lipids, or metals may improve drug bioavailability, enabling slow release and reducing adverse effects.
Gene Therapy Recombinant adeno-associated virus (AAV) vector-mediated gene therapy targeting prostaglandin F2α synthesis in the AC [97] Intravitreal injections of AAV-F-iTrkB (AAV farnesylation of the intracellular domain of TrkB) [98]	Preclinical	Ocular gene therapy can target the TM to increase AH outflow and offer neuroprotection by limiting retinal ganglion cell (RGC) loss.
Stem Cell Applications Bone marrow-derived Mesenchymal Stem Cells (MSCs) injected into the AC [99] Human-induced pluripotent stem cells-derived RGCs [100] Human adipose-derived MSCs conditioned-medium ocular instillation [101]	Preclinical	Stem cells can be used to improve TM structure and function, promote RGC survival, and improve corneal barrier dysfunction.
Umbilical Cord Blood Serum (CBS) Eye Drops A. Singapore Cord Blood Bank CBS eye drops (Singapore National Eye Center) [102]	Phase 2	CBS drops contain high levels of growth factors and anti-inflammatory cytokines.
Acupuncture A. Niemtzow Acupuncture Protocol (University of Pittsburgh, USA) [103]	Phase 3	Acupuncture may downregulate proinflammatory cytokines and increase the release of acetylcholine in the lacrimal glands, promoting tear secretion.

Legend: TODDD^®^ = Topical Ophthalmic Drug Delivery Device^®^, PDCLs = passive diffusion contact lenses, CLs = contact lenses, MICLs = molecular imprinted contact lenses, PPs = punctual plugs, AC = anterior chamber, II = intracameral implants, TM = trabecular meshwork, SI = subconjunctival implants, IPL = intense pulsed light, TPD = thermal pulsation devices, AAV = adeno-associated virus, AH = aqueous humor, RGC = retinal ganglion cell, CBS = cord blood serum.

## Data Availability

No new data were created or analyzed in this study. Data sharing is not applicable to this article.

## References

[1] Tham Y.C., Li X., Wong T.Y., Quigley H.A., Aung T., Cheng C.Y. (2014). Global prevalence of glaucoma and projections of glaucoma burden through 2040: A systematic review and meta-analysis. Ophthalmology.

[2] Pooja Prajwal M.R., Gopalakrishna H.N., Kateel R. (2013). An exploratory study on the drug utilization pattern in glaucoma patients at a tertiary care hospital. J. App. Pharm. Sci..

[3] Craig J.P., Nichols K.K., Akpek E.K., Caffery B., Dua H.S., Joo C.K., Liu Z., Nelson J.D., Nichols J.J., Tsubota K. (2017). TFOS DEWS II definition and classification report. Ocul. Surf..

[4] Gomes J.A.P., Azar D.T., Baudouin C., Efron N., Hirayama M., Horwath-Winter J., Kim T., Mehta J.S., Messmer E.M., Pepose J.S. (2017). TFOS DEWS II iatrogenic report. Ocul. Surf..

[5] Ruiz-Lozano R.E., Azar N.S., Mousa H.M., Quiroga-Garza M.E., Komai S., Wheelock-Gutierrez L., Cartes C., Perez V.L. (2023). Ocular surface disease: A known yet overlooked side effect of topical glaucoma therapy. Front. Toxicol..

[6] Baudouin C., Liang H., Hamard P., Riancho L., Creuzot-Garcher C., Warnet J.M., Brignole-Baudouin F. (2008). The ocular surface of glaucoma patients treated over the long term expresses inflammatory markers related to both T-helper 1 and T-helper 2 pathways. Ophthalmology.

[7] Roda M., Corazza I., Bacchi Reggiani M.L., Pellegrini M., Taroni L., Giannaccare G., Versura P. (2020). Dry eye disease and tear cytokine levels-a meta-analysis. Int. J. Mol. Sci..

[8] Roy N.S., Wei Y., Ying G.S., Maguire M.G., Asbell P.A. (2023). Association of tear cytokine concentrations with symptoms and signs of dry eye disease: Baseline data from the Dry Eye Assessment and Management (DREAM) study. Curr. Eye Res..

[9] Scarpellini C., Ramos Llorca A., Lanthier C., Klejborowska G., Augustyns K. (2023). The potential role of regulated cell death in dry eye diseases and ocular surface dysfunction. Int. J. Mol. Sci..

[10] Fineide F., Magnø M., Dahlø K., Kolko M., Heegaard S., Vehof J., Utheim T.P. (2024). Topical glaucoma medications-possible implications on the meibomian glands. Acta Ophthalmol..

[11] Kolko M., Gazzard G., Baudouin C., Beier S., Brignole-Baudouin F., Cvenkel B., Fineide F., Hedengran A., Hommer A., Jespersen E. (2023). Impact of glaucoma medications on the ocular surface and how ocular surface disease can influence glaucoma treatment. Ocul. Surf..

[12] Li G., Akpek E.K., Ahmad S. (2022). Glaucoma and ocular surface disease: More than meets the eye. Clin. Ophthalmol..

[13] Zhang X., Vadoothker S., Munir W.M., Saeedi O. (2019). Ocular surface disease and glaucoma medications: A clinical approach. Eye Contact Lens.

[14] Garcia-Terraza A.L., Jimenez-Collado D., Sanchez-Sanoja F., Arteaga-Rivera J.Y., Morales Flores N., Pérez-Solórzano S., Garfias Y., Graue-Hernández E.O., Navas A. (2022). Reliability, repeatability, and accordance between three different corneal diagnostic imaging devices for evaluating the ocular surface. Front. Med..

[15] Schmidl D., Schlatter A., Chua J., Tan B., Garhöfer G., Schmetterer L. (2020). Novel approaches for imaging-based diagnosis of ocular surface disease. Diagnostics.

[16] Andole S., Senthil S. (2023). Ocular surface disease and anti-glaucoma medications: Various features, diagnosis, and management guidelines. Semin. Ophthalmol..

[17] Scelfo C., ElSheikh R.H., Shamim M.M., Abbasian J., Ghaffarieh A., Elhusseiny A.M. (2023). Ocular surface disease in glaucoma patients. Curr. Eye Res..

[18] Cochrane Library What Is PICO?. https://www.cochranelibrary.com/about-pico.

[19] Harzing A.W. Publish or Perish. https://harzing.com/resources/publish-or-perish.

[20] Seider N., Miller B., Beiran I. (2008). Topical glaucoma therapy as a risk factor for nasolacrimal duct obstruction. Am. J. Ophthalmol..

[21] Kuppens E.V., de Jong C.A., Stolwijk T.R., de Keizer R.J., van Best J.A. (1995). Effect of timolol with and without preservative on the basal tear turnover in glaucoma. Br. J. Ophthalmol..

[22] Yuan X., Ma X., Yang L., Zhou Q., Li Y. (2021). β-blocker eye drops affect ocular surface through β2 adrenoceptor of corneal limbal stem cells. BMC Ophthalmol..

[23] Mastropasqua R., Agnifili L., Fasanella V., Curcio C., Brescia L., Lanzini M., Fresina M., Mastropasqua L., Marchini G. (2015). Corneoscleral limbus in glaucoma patients: In vivo confocal microscopy and immunocytological study. Investig. Ophthalmol. Vis. Sci..

[24] Inoue K., Okugawa K., Kato S., Inoue Y., Tomita G., Oshika T., Amano S. (2003). Ocular factors relevant to anti-glaucomatous eyedrop-related keratoepitheliopathy. J. Glaucoma.

[25] Zhou X., Zhang X., Zhou D., Zhao Y., Duan X. (2022). A narrative review of ocular surface disease related to anti-glaucomatous medications. Ophthalmol. Ther..

[26] Rolle T., Spinetta R., Nuzzi R. (2017). Long term safety and tolerability of Tafluprost 0.0015% vs. Timolol 0.1% preservative-free in ocular hypertensive and in primary open-angle glaucoma patients: A cross sectional study. BMC Ophthalmol..

[27] Russ H.H., Costa V.P., Ferreira F.M., Valgas S.R., Correa Neto M.A., Strobel E., Truppel J.H. (2007). Conjunctival changes induced by prostaglandin analogues and timolol maleate: A histomorphometric study. Arq. Bras. Oftalmol..

[28] Yoshino T., Fukuchi T., Togano T., Seki M., Ikegaki H., Abe H. (2013). Eyelid and eyelash changes due to prostaglandin analog therapy in unilateral treatment cases. Jpn. J. Ophthalmol..

[29] Yamada H., Yoneda M., Gosho M., Kato T., Zako M. (2016). Bimatoprost, latanoprost, and tafluprost induce differential expression of matrix metalloproteinases and tissue inhibitor of metalloproteinases. BMC Ophthalmol..

[30] Mocan M.C., Uzunosmanoglu E., Kocabeyoglu S., Karakaya J., Irkec M. (2016). The association of chronic topical prostaglandin analog use with meibomian gland dysfunction. J. Glaucoma.

[31] Yeh P.H., Cheng Y.C., Shie S.S., Lee Y.S., Shen S.C., Chen H.S., Wu W.C., Su W.W. (2021). Brimonidine related acute follicular conjunctivitis: Onset time and clinical presentations, a long-term follow-up. Medicine.

[32] Trotta D., Zucchelli M., Salladini C., Ballerini P., Rossi C., Aricò M. (2024). Brimonidine eye drops within the reach of children: A possible foe. Children.

[33] Rohrschneider K., Koch H.-R. (1991). [Effects of acetazolamide (Diamox^®^, Glaupax^®^) on tear production]. Klin. Monatsblätter für Augenheilkd. [Clin. Mon. Newslett. Ophthalmol.].

[34] Terai N., Müller-Holz M., Spoerl E., Pillunat L.E. (2011). Short-term effect of topical antiglaucoma medication on tear-film stability, tear secretion, and corneal sensitivity in healthy subjects. Clin. Ophthalmol..

[35] Skaat A., Rosman M.S., Chien J.L., Mogil R.S., Ren R., Liebmann J.M., Ritch R., Park S.C. (2016). Effect of pilocarpine hydrochloride on the schlemm canal in healthy eyes and eyes with open-angle glaucoma. JAMA Ophthalmol..

[36] Zhang Y., Kam W.R., Liu Y., Chen X., Sullivan D.A. (2017). Influence of pilocarpine and timolol on human meibomian gland epithelial cells. Cornea.

[37] Hartenbaum D., Maloney S., Vaccarelli L., Liss C., Wilson H., Gormley G.J. (1999). Comparison of dorzolamide and pilocarpine as adjunctive therapy in patients with open-angle glaucoma and ocular hypertension. Clin. Ther..

[38] Mehran N.A., Sinha S., Razeghinejad R. (2020). New glaucoma medications: Latanoprostene bunod, netarsudil, and fixed combination netarsudil-latanoprost. Eye.

[39] Patton G.N., Lee H.J. (2024). Chemical insights into topical agents in intraocular pressure management: From glaucoma etiopathology to therapeutic approaches. Pharmaceutics.

[40] Srivastava K., Bhatnagar K.R., Shakrawal J., Tandon M., Jaisingh K., Pandey L., Roy F. (2024). Ocular surface changes in primary open-angle glaucoma on anti-glaucoma medications versus treatment-naïve patients. Indian J. Ophthalmol..

[41] Ye Y., Xu Y., Yang Y., Fan Y., Liu P., Yu K., Yu M. (2022). Wide corneal epithelial thickness mapping in eyes with topical antiglaucoma therapy using optical coherence tomography. Transl. Vis. Sci. Technol..

[42] Pai V., Reddy L.S.H. (2018). Prevalence of ocular surface disease in patients with glaucoma on topical medications. Asian J. Ophthalmol..

[43] Pérez-Bartolomé F., Martínez-de-la-Casa J.M., Arriola-Villalobos P., Fernández-Pérez C., Polo V., García-Feijoó J. (2017). Ocular surface disease in patients under topical treatment for glaucoma. Eur. J. Ophthalmol..

[44] Cvenkel B., Štunf Š., Srebotnik Kirbiš I., Strojan Fležar M. (2015). Symptoms and signs of ocular surface disease related to topical medication in patients with glaucoma. Clin. Ophthalmol..

[45] Saade C.E., Lari H.B., Berezina T.L., Fechtner R.D., Khouri A.S. (2015). Topical glaucoma therapy and ocular surface disease: A prospective, controlled cohort study. Can. J. Ophthalmol..

[46] Van Went C., Alalwani H., Brasnu E., Pham J., Hamard P., Baudouin C., Labbé A. (2011). [Corneal sensitivity in patients treated medically for glaucoma or ocular hypertension]. J. Fr. Ophtalmol..

[47] Fechtner R.D., Godfrey D.G., Budenz D., Stewart J.A., Stewart W.C., Jasek M.C. (2010). Prevalence of ocular surface complaints in patients with glaucoma using topical intraocular pressure-lowering medications. Cornea.

[48] Jaenen N., Baudouin C., Pouliquen P., Manni G., Figueiredo A., Zeyen T. (2007). Ocular symptoms and signs with preserved and preservative-free glaucoma medications. Eur. J. Ophthalmol..

[49] Pisella P.J., Pouliquen P., Baudouin C. (2002). Prevalence of ocular symptoms and signs with preserved and preservative free glaucoma medication. Br. J. Ophthalmol..

[50] Petounis A.D., Akritopoulos P. (1989). Influence of topical and systemic beta-blockers on tear production. Int. Ophthalmol..

[51] Kaur I.P., Lal S., Rana C., Kakkar S., Singh H. (2009). Ocular preservatives: Associated risks and newer options. Cutan. Ocul. Toxicol..

[52] Goldstein M.H., Silva F.Q., Blender N., Tran T., Vantipalli S. (2022). Ocular benzalkonium chloride exposure: Problems and solutions. Eye.

[53] Debbasch C., Brignole F., Pisella P.J., Warnet J.M., Rat P., Baudouin C. (2001). Quaternary ammoniums and other preservatives’ contribution in oxidative stress and apoptosis on Chang conjunctival cells. Investig. Ophthalmol. Vis. Sci..

[54] Zhang R., Park M., Richardson A., Tedla N., Pandzic E., de Paiva C.S., Watson S., Wakefield D., Di Girolamo N. (2020). Dose-dependent benzalkonium chloride toxicity imparts ocular surface epithelial changes with features of dry eye disease. Ocul. Surf..

[55] Ramli N., Supramaniam G., Samsudin A., Juana A., Zahari M., Choo M.M. (2015). Ocular surface disease in glaucoma: Effect of polypharmacy and preservatives. Optom. Vis. Sci..

[56] Sherwood M.B., Grierson I., Millar L., Hitchings R.A. (1989). Long-term morphologic effects of antiglaucoma drugs on the conjunctiva and Tenon’s capsule in glaucomatous patients. Ophthalmology.

[57] Kumar S., Singh T., Ichhpujani P., Vohra S. (2019). Ocular surface disease with BAK preserved travoprost and polyquaternium 1(Polyquad) preserved travoprost. Rom. J. Ophthalmol..

[58] Ammar D.A., Kahook M.Y. (2011). Effects of benzalkonium chloride- or polyquad-preserved fixed combination glaucoma medications on human trabecular meshwork cells. Mol. Vis..

[59] Katz L.J. (2002). Twelve-month evaluation of brimonidine-purite versus brimonidine in patients with glaucoma or ocular hypertension. J. Glaucoma.

[60] Ryan G., Fain J.M., Lovelace C., Gelotte K.M. (2011). Effectiveness of ophthalmic solution preservatives: A comparison of latanoprost with 0.02% benzalkonium chloride and travoprost with the sofZia preservative system. BMC Ophthalmol..

[61] Kanamoto T., Kiuchi Y., Tanito M., Mizoue S., Naito T., Teranishi S., Hirooka K., Rimayanti U. (2015). Comparison of the toxicity profile of benzalkonium chloride-preserved tafluprost and SofZia-preserved travoprost applied to the ocular surface. J. Ocul. Pharmacol. Ther..

[62] Moiseev R.V., Morrison P.W.J., Steele F., Khutoryanskiy V.V. (2019). Penetration enhancers in ocular drug delivery. Pharmaceutics.

[63] Uusitalo H., Chen E., Pfeiffer N., Brignole-Baudouin F., Kaarniranta K., Leino M., Puska P., Palmgren E., Hamacher T., Hofmann G. (2010). Switching from a preserved to a preservative-free prostaglandin preparation in topical glaucoma medication. Acta Ophthalmol..

[64] Iester M., Telani S., Frezzotti P., Motolese I., Figus M., Fogagnolo P., Perdicchi A. (2014). Ocular surface changes in glaucomatous patients treated with and without preservatives beta-blockers. J. Ocul. Pharmacol. Ther..

[65] Jandroković S., Vidas Pauk S., Lešin Gaćina D., Skegro I., Tomić M., Masnec S., Kuzman T., Kalauz M. (2022). Tolerability in glaucoma patients switched from preserved to preservative-free prostaglandin-timolol combination: A prospective real-life study. Clin. Ophthalmol..

[66] Wolffsohn J.S., Arita R., Chalmers R., Djalilian A., Dogru M., Dumbleton K., Gupta P.K., Karpecki P., Lazreg S., Pult H. (2017). TFOS DEWS II diagnostic methodology report. Ocul. Surf..

[67] Jones L., Downie L.E., Korb D., Benitez-Del-Castillo J.M., Dana R., Deng S.X., Dong P.N., Geerling G., Hida R.Y., Liu Y. (2017). TFOS DEWS II management and therapy report. Ocul. Surf..

[68] Kim J.G., An J.H., Cho S.Y., Lee C.E., Shim K.Y., Jun J.H. (2023). Efficacy of topical 0.05% cyclosporine A for ocular surface disease related to topical anti-glaucoma medications. J. Ocul. Pharmacol. Ther..

[69] Pleyer U., Ursell P.G., Rama P. (2013). Intraocular pressure effects of common topical steroids for post-cataract inflammation: Are they all the same?. Ophthalmol. Ther..

[70] Giannaccare G., Pellegrini M., Sebastiani S., Bernabei F., Roda M., Taroni L., Versura P., Campos E.C. (2019). Efficacy of omega-3 fatty acid supplementation for treatment of dry eye disease: A meta-analysis of randomized clinical trials. Cornea.

[71] Cui X., Xiang J., Zhu W., Wei A., Le Q., Xu J., Zhou X. (2016). Vitamin A palmitate and carbomer gel protects the conjunctiva of patients with long-term prostaglandin analogs application. J. Glaucoma.

[72] Vazirani J., Sridhar U., Gokhale N., Doddigarla V.R., Sharma S., Basu S. (2023). Autologous serum eye drops in dry eye disease: Preferred practice pattern guidelines. Indian J. Ophthalmol..

[73] McDonald M., Janik S.B., Bowden F.W., Chokshi A., Singer M.A., Tighe S., Mead O.G., Nanda S., Qazi M.A., Dierker D. (2023). Association of treatment duration and clinical outcomes in dry eye treatment with sutureless cryopreserved amniotic membrane. Clin. Ophthalmol..

[74] Conlon R., Saheb H., Ahmed I.I.K. (2017). Glaucoma treatment trends: A review. Can. J. Ophthalmol..

[75] Al-Qaysi Z.K., Beadham I.G., Schwikkard S.L., Bear J.C., Al-Kinani A.A., Alany R.G. (2023). Sustained release ocular drug delivery systems for glaucoma therapy. Expert Opin. Drug Deliv..

[76] M Grover L., Moakes R., Rauz S. (2022). Innovations in fluid-gel eye drops for treating disease of the eye: Prospects for enhancing drug retention and reducing corneal scarring. Expert Rev. Ophthalmol..

[77] Brandt J.D., Sall K., DuBiner H., Benza R., Alster Y., Walker G., Semba C.P. (2016). Six-month intraocular pressure reduction with a topical bimatoprost ocular insert: Results of a phase II randomized controlled study. Ophthalmology.

[78] Kesav N.P., Young C.E.C., Ertel M.K., Seibold L.K., Kahook M.Y. (2021). Sustained-release drug delivery systems for the treatment of glaucoma. Int. J. Ophthalmol..

[79] Hsu K.H., Carbia B.E., Plummer C., Chauhan A. (2015). Dual drug delivery from vitamin E loaded contact lenses for glaucoma therapy. Eur. J. Pharm. Biopharm..

[80] Ciolino J.B., Ross A.E., Tulsan R., Watts A.C., Wang R.F., Zurakowski D., Serle J.B., Kohane D.S. (2016). Latanoprost-eluting contact lenses in glaucomatous monkeys. Ophthalmology.

[81] Anirudhan T.S., Nair A.S., Parvathy J. (2016). Extended wear therapeutic contact lens fabricated from timolol imprinted carboxymethyl chitosan-g-hydroxy ethyl methacrylate-g-poly acrylamide as a onetime medication for glaucoma. Eur. J. Pharm. Biopharm..

[82] Clinical Trials. Safety and Intraocular Lowering Effect of Delivery of Travoprost Evolute® in Subjects with Elevated Intraocular Pressure. https://clinicaltrials.gov/study/NCT04962009?term=EVOLUTE&rank=2.

[83] Perera S.A., Ting D.S., Nongpiur M.E., Chew P.T., Aquino M.C., Sng C.C., Ho S.W., Aung T. (2016). Feasibility study of sustained-release travoprost punctum plug for intraocular pressure reduction in an Asian population. Clin. Ophthalmol..

[84] Weinreb R.N., Bacharach J., Brubaker J.W., Medeiros F.A., Bejanian M., Bernstein P., Robinson M.R. (2023). Bimatoprost implant biodegradation in the Phase 3, randomized, 20-month ARTEMIS studies. J. Ocul. Pharmacol. Ther..

[85] Clinical Trials. Safety and Efficacy of ENV515 Travoprost Extended Release (XR) in Patients with Bilateral Ocular Hypertension or Primary Open Angle Glaucoma. https://clinicaltrials.gov/study/NCT02371746?term=ENV515&rank=1.

[86] Clinical Trials. Safety, and Efficacy of OTX-TIC in Participants with Open Angle Glaucoma or Ocular Hypertension. https://clinicaltrials.gov/study/NCT04360174?term=OTX-TIC&rank=1.

[87] Berdahl J.P., Sarkisian S.R., Ang R.E., Doan L.V., Kothe A.C., Usner D.W., Katz L.J., Navratil T. (2024). Efficacy and safety of the travoprost intraocular implant in reducing topical iop-lowering medication burden in patients with open-angle glaucoma or ocular hypertension. Drugs.

[88] Rafiei F., Tabesh H., Farzad F. (2020). Sustained subconjunctival drug delivery systems: Current trends and future perspectives. Int. Ophthalmol..

[89] Safir M., Twig G., Mimouni M. (2024). Dry eye disease management. BMJ.

[90] Miao S., Yan R., Jia Y., Pan Z. (2022). Effect of intense pulsed light therapy in dry eye disease caused by meibomian gland dysfunction: A systematic review and meta-analysis. Eye Contact Lens.

[91] Hu J., Zhu S., Liu X. (2022). Efficacy and safety of a vectored thermal pulsation system (Lipiflow^®^) in the treatment of meibomian gland dysfunction: A systematic review and meta-analysis. Graefes. Arch. Clin. Exp. Ophthalmol..

[92] Antwi A., Schill A.W., Redfern R., Ritchey E.R. (2024). Effect of low-level light therapy in individuals with dry eye disease. Ophthalmic Physiol. Opt..

[93] Clinical Trials. Photobiomodulation with REd vs. BluE Light (REBEL). https://clinicaltrials.gov/study/NCT06371300.

[94] Ciociola E.C., Fernandez E., Kaufmann M., Klifto M.R. (2024). Future directions of glaucoma treatment: Emerging gene, neuroprotection, nanomedicine, stem cell, and vascular therapies. Curr. Opin. Ophthalmol..

[95] Occhiutto M.L., Maranhão R.C., Costa V.P., Konstas A.G. (2020). Nanotechnology for medical and surgical glaucoma therapy-a review. Adv. Ther..

[96] Maulvi F.A., Patil R.J., Desai A.R., Shukla M.R., Vaidya R.J., Ranch K.M., Vyas B.A., Shah S.A., Shah D.O. (2019). Effect of gold nanoparticles on timolol uptake and its release kinetics from contact lenses: In vitro and in vivo evaluation. Acta Biomater..

[97] Chern K.J., Nettesheim E.R., Reid C.A., Li N.W., Marcoe G.J., Lipinski D.M. (2022). Prostaglandin-based rAAV-mediated glaucoma gene therapy in Brown Norway rats. Commun. Biol..

[98] Nishijima E., Honda S., Kitamura Y., Namekata K., Kimura A., Guo X., Azuchi Y., Harada C., Murakami A., Matsuda A. (2023). Vision protection and robust axon regeneration in glaucoma models by membrane-associated Trk receptors. Mol. Ther..

[99] Roubeix C., Godefroy D., Mias C., Sapienza A., Riancho L., Degardin J., Fradot V., Ivkovic I., Picaud S., Sennlaub F. (2015). Intraocular pressure reduction and neuroprotection conferred by bone marrow-derived mesenchymal stem cells in an animal model of glaucoma. Stem Cell Res. Ther..

[100] Vrathasha V., Nikonov S., Bell B.A., He J., Bungatavula Y., Uyhazi K.E., Murthy Chavali V.R. (2022). Transplanted human induced pluripotent stem cells- derived retinal ganglion cells embed within mouse retinas and are electrophysiologically functional. iScience.

[101] Imaizumi T., Hayashi R., Kudo Y., Li X., Yamaguchi K., Shibata S., Okubo T., Ishii T., Honma Y., Nishida K. (2023). Ocular instillation of conditioned medium from mesenchymal stem cells is effective for dry eye syndrome by improving corneal barrier function. Sci. Rep..

[102] Wong J., Govindasamy G., Prasath A., Hwang W., Ho A., Yeo S., Tong L. (2023). Allogeneic umbilical cord plasma eyedrops for the treatment of recalcitrant dry eye disease patients. J. Clin. Med..

[103] Prinz J., Maffulli N., Fuest M., Walter P., Hildebrand F., Migliorini F. (2022). Acupuncture for the management of dry eye disease. Front. Med..

